# Giant Cell Tumor of the Clivus in a 19-Year-Old Male Presenting With Doubling of Vision: A Case Report

**DOI:** 10.7759/cureus.59117

**Published:** 2024-04-26

**Authors:** Ma. Regina D Matabang, Paul Vincent A Opinaldo

**Affiliations:** 1 Center for Neurological Sciences, Quirino Memorial Medical Center, Quezon City, PHL

**Keywords:** transphenoidal surgery, skull mass, bone tumor, brain tumor, clivus, giant cell tumor

## Abstract

Giant cell tumor (GCT) of the skull is an extremely rare condition, accounting for less than one percent of all bone GCTs. Clival GCT is even rarer, with only 25 cases documented to date. It generally follows a benign course; however, due to its location and vascularity, it can be locally aggressive. Complete resection of GCT in this location may be challenging, resulting in residual tumors. In this paper, we report a case of a 19-year-old male who presented with a chronic headache later accompanied by diplopia and was noted to have a mass spanning the sella and the clivus on cranial imaging. The histopathology report of the excised mass revealed findings compatible with GCT of the bone. Most GCTs remain stable in the first two years after initial treatment. However, four months after its partial excision, the clival GCT continued to progress. The patient underwent adjuvant radiation therapy, yet symptoms persisted. This profile highlights the crucial role of long-term surveillance and prompt adjuvant radiation therapy and chemotherapy.

## Introduction

Giant cell tumor (GCT) of the bone accounts for 2.4-5% of all primary bone tumors in adults. This most commonly occurs in the epiphyses of long bones, such as the distal femur, proximal tibia, and distal radius. In 1-2% of cases, however, the tumor can be found in the skull, particularly in the ethmoid, sphenoid, and petrous temporal bones [1-3]. Since 1983, a few reported cases of GCT of the clivus have been reported. The exact etiology of GCTs, in general, has yet to be fully understood. The Receptor Activator of Nuclear Factor Kappa beta (RANK) pathway has been implicated, which plays a role in the differentiation of precursor cells into multinucleated osteoclasts and the activation of osteoclasts for bone resorption [3].

The main presentation in most cases of GCT of the clivus includes headache owing to increased intracranial pressure and diplopia caused by abducens nerve palsy [1]. Its location and the risk of injury to the surrounding neurovascular structures are the key factors that render complete resection difficult. Residual mass, therefore, results in an increased rate of recurrence. 

In this report, we present a case of a 19-year-old male who had a history of chronic headache and diplopia and was eventually diagnosed with GCT of the clivus.

## Case presentation

This is a case of a 19-year-old male who, eight months before admission, experienced a sudden left temporal headache, non-radiating, graded 5/10, with no other accompanying symptoms. This spontaneously resolved after thirty minutes of resting. A headache of the same characteristic was noted to be present daily, more prominent upon waking up, when straining, coughing, and sneezing. He was still able to do his activities of daily living. 

Five months before admission, there was a persistent headache of the same characteristics, this time accompanied by diplopia on leftward gaze and vomiting. 

**Figure 1:**
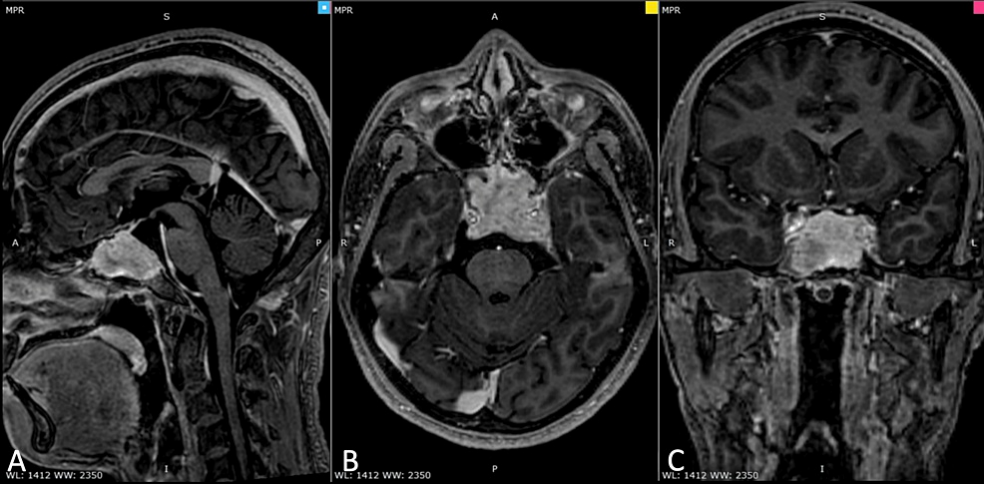
Pre-operative brain MRI T1 with contrast showing an avidly enhancing, lobulated, irregularly shaped sellar mass measuring 3.8 x 3.3 x 2.3 cm (AP x W x CC) extending superiorly and inferiorly, eroding the dorsum of the sella and the upper clivus. (A) Sagittal view, (B) Axial view, and (C) Coronal view.

Four months before admission, he noted parosmia and worsening visual disturbance in the left eye. A brain MRI with contrast was subsequently done (Figure 1). This showed an avidly enhancing, lobulated, irregularly shaped sellar mass measuring 3.8 x 3.3 x 2.3 cm (AP x W x CC) extending superiorly and inferiorly, eroding the dorsum of the sella and the upper clivus. There were no signs of increased intracranial pressure, such as ventricular dilatation and transependymal seepage. He was then started on dexamethasone 5 mg/tablet, one tablet every six hours, and was subsequently admitted for surgery. On physical examination, he had a Glasgow Coma Scale (GCS) score of 15, with no neurologic deficits. Cranial nerve palsies causing diplopia are likely to resolve after dexamethasone administration.

**Figure 2:**
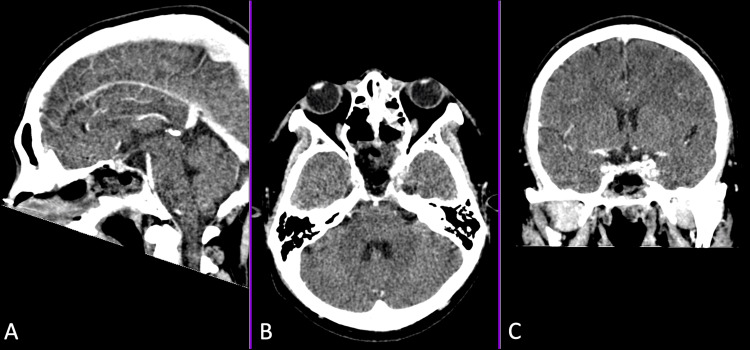
Postoperative head CT with contrast showed a slight decrease in the bulk of the clival mass to 3.2 x 3.0 x 1.0 cm (AP x W x CC). (A) Sagittal view, (B) Axial view, and (C) Coronal view.

**Figure 3:**
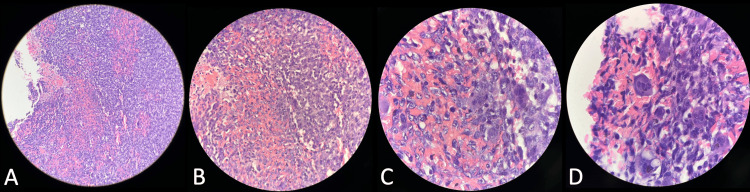
Routine histopathology showed a giant cell-rich lesion, which could either be GCT of the bone or a central giant cell granuloma. Microsections disclose a cellular lesion consisting of a proliferation of mononuclear ovoid to spindle-shaped cells associated with multinucleated osteoclast-type giant cells and varying amounts of collagen. Focal hemorrhagic areas are also noted. These giant cells have a variable number of nuclei, an ill-defined cell membrane, pale eosinophilic cytoplasm, vesicular nuclei, and occasionally prominent small nucleoli. There is focally increased mitotic activity (up to 8–10 hpf). Magnification: (A) scanner, (B) low power objective, (C) high power objective, and (D) oil immersion objective. GCT: giant cell tumor.

**Figure 4:**
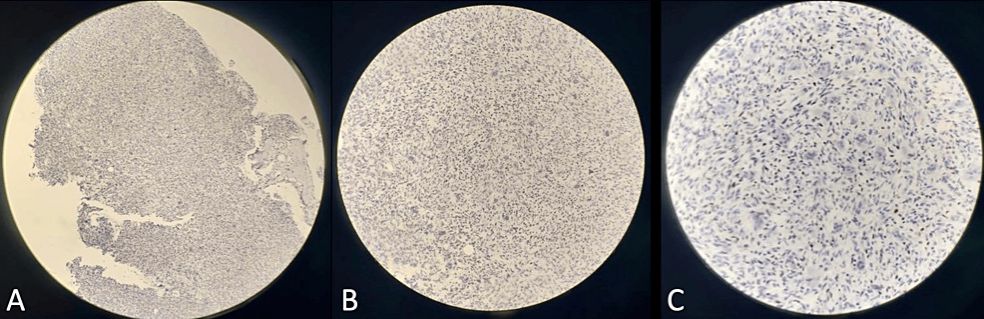
An immunohistochemistry study on the specimen later showed positivity for p63. Magnification: (A) scanner, (B) low power objective, and (C) high power objective.

He underwent an endoscopic endonasal approach for uncinectomy, antrostomy, anterior and posterior ethmoidectomy, and middle turbinectomy with subtotal excision of the clival tumor, which was tolerated well. Postoperatively, he was status-quo neurologically. Postoperative head CT scan with contrast showed a slight decrease in the bulk of heterogeneously enhancing clival mass measuring 3.2 x 3.0 x 1.0 cm (AP x W x CC) (Figure 2). The histopathology report initially showed a giant cell-rich lesion, which could either be GCT of the bone or central giant cell granuloma (Figure 3). An immunohistochemistry study on the specimen later showed positivity for p63 and confirmed the diagnosis of GCT of the clivus (Figure 4). He was eventually discharged on the ninth postoperative day. 

**Figure 5:**
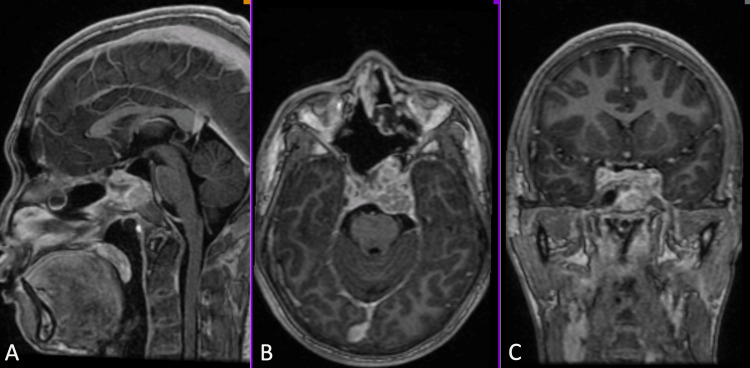
Postoperative brain MRI T1 with contrast showed a heterogeneously enhancing, lobulated mass in the clivus measuring 3.8 x 4.1 x 2.7 cm (AP x W x CC). (A) Sagittal view, (B) Axial view, and (C) Coronal view.

On follow-up of this patient four months after treatment, brain MRI with contrast revealed a heterogeneously enhancing, lobulated mass on the clivus measuring 3.8 cm x 4.1 cm x 2.7 cm with extension to the sphenoid body, left sphenoid sinus, sella turcica, cavernous sinus, and medial aspect of the left middle cranial fossa (Figure 5). The patient then underwent intensity-modulated radiation therapy (IMRT), with a total dose of 50 Gy given over 25 sessions (2 Gy/session, five sessions/week over five weeks). 

**Figure 6:**
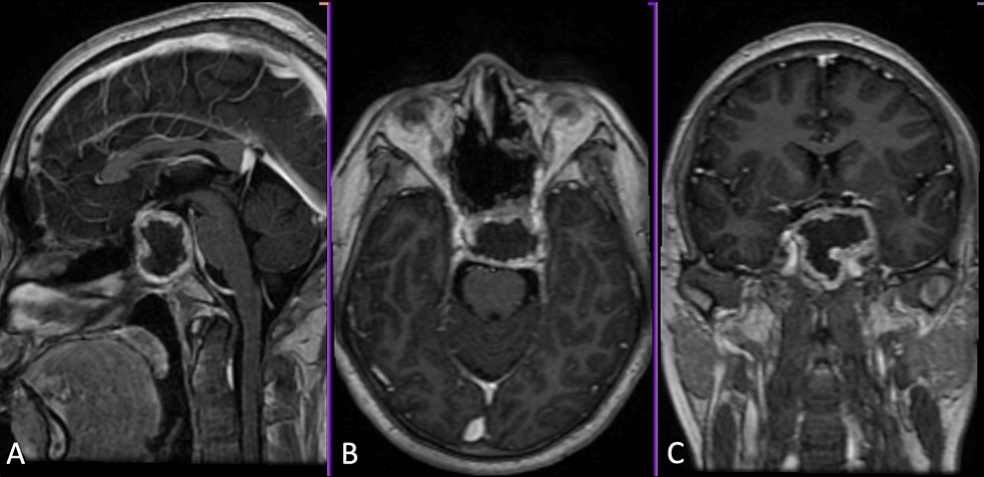
Post-radiation therapy, brain MRI T1 with contrast showed an interval decrease in the size of the mass, measuring 2.7 x 3.8 x 3.7 cm (AP x W x CC), which now demonstrates intralesional necrosis. (A) Sagittal view, (B) Axial view, and (C) Coronal view.

A surveillance brain MRI with contrast was done four months post-radiation therapy due to persistent headaches (Figure 6). It revealed an interval decrease in the size of the mass measuring 2.7 x 3.8 x 3.7 cm, now demonstrating intralesional necrosis compared to the pre-radiation MRI. He was prescribed dexamethasone 4 mg/tablet, one tablet every 12 hours, which provided partial and temporary relief.

As of the time of writing, six months post-radiation, the patient is still experiencing intermittent headaches. Re-operation has been discussed. However, due to limited chances for gross total excision owing to the location of the mass and its extent, the patient still refused to undergo surgery. Further management being pursued is chemotherapy through denosumab.

## Discussion

GCT of the bone accounts for 2.4-5% of all primary bone tumors in adults. Approximately half of the cases manifest in young adults in their third to fourth decades. This most commonly occurs in the epiphyses of long bones, such as the distal femur, proximal tibia, and distal radius [4]. Rarely, it affects the skull, with a propensity to occur in the ethmoid, sphenoid, and petrous temporal bones. GCT of the clivus is extremely rare, with only a few cases reported to date, including this case [2].

The exact etiology of GCTs is unknown but is presumed to be due to giant cell formation by activated spindle-like stromal cells. The interaction among chemotactic factors such as interleukins (IL) -6, -8, -11, -17, and -34, tumor necrosis factor, basic fibroblast growth factor, vascular endothelial growth factor, macrophage colony-stimulating factor, RANKL, and cathepsin K; chemokines IL-8 and TGF-B1; and enzymes matrix metalloproteinases (MMP)-9 and -13 serve to differentiate monocytes into macrophages. These macrophages that have acquired osteoclastic activity are responsible for the bone resorption observed within the lesion. In addition, giant cells that have increased expression of the RANK receptor, a key mediator of osteoclastogenesis, and activation of RANKL secreted by stromal cells lead to further bone resorption. The activated osteoclasts release tumor growth factors that propagate this vicious tumor/bone cycle [2,4].

GCTs usually follow a benign course, but malignant transformation has been reported [5]. They can be locally aggressive, causing bone erosions due to the mechanisms previously discussed [5]. The majority of patients with clival GCTs presented with headaches and diplopia. Headache is caused by the involvement of the dura around the clivus, which may be compounded, in some cases, by increased intracranial pressure secondary to hydrocephalus. Diplopia is caused by compression of the abducens nerve as it passes through the Dorello's canal or the cavernous sinus. 

GCTs of the skull, especially that of the clivus, prove to be challenging in terms of treatment. Due to its location and vascularity, complete resection of the mass is difficult, resulting in residual or recurrent tumors. In more extensive lesions, bony erosion of the mass can also increase the incidence of cerebrospinal fluid leak due to its proximity to the prepontine cistern, hence leading to higher postoperative complications. Recurrence could occur within the first two years following treatment, making long-term surveillance necessary [6]. Because of their rarity and lack of available literature, the definitive treatment of clival GCTs has not been standardized. In most cases, the mainstay of therapy is function-preserving surgery combined with adjuvant chemoradiation. In a case of residual clival GCT reported by Huh et al. in 2018, the patient was treated with a combination of adjuvant denosumab therapy, a monoclonal antibody that targets RANK-L, and proton therapy at 45-60 Gy doses [3,6].

In the case of our patient, who presented with headache and diplopia over several months, surgical excision of the clival mass by endoscopic endonasal approach was done, later revealing GCT on histopathologic analysis. Congruent with reports, there was incomplete resection of the mass in an attempt to preserve the surrounding structures. On follow-up four months after surgical intervention, there was a recurrence of headaches, probably due to meningeal involvement of the clivus. Surveillance neuroimaging revealed progression in the size and extent of the clival mass. The patient then underwent radiotherapy. Follow-up cranial imaging after radiotherapy showed a decrease in the lesion size to 2.7 x 3.8 x 3.7 cm (AP x W x CC) with intralesional necrosis. However, despite radiation therapy and Paracetamol, the patient’s headache persisted. Further treatment with chemotherapy, such as denosumab, is being pursued; however, it has not yet been given due to financial limitations. 

## Conclusions

Clival GCTs are exceedingly rare. Complete resection of these tumors proves challenging because of their location and vascularity. The risk of progression is thereby increased remarkably during the first two years after initial treatment. This highlights the need for long-term surveillance and prompt management of patients with residual tumors. Apart from surgical intervention, the use of adjuvant radiotherapy and chemotherapy, such as denosumab, may be considered. Further studies on the standard treatment of clival GCTs are recommended.
